# IL-27R signaling controls myeloid cells accumulation and antigen-presentation in atherosclerosis

**DOI:** 10.1038/s41598-017-01828-8

**Published:** 2017-05-23

**Authors:** Iuliia O. Peshkova, Aliia R. Fatkhullina, Zbigniew Mikulski, Klaus Ley, Ekaterina K. Koltsova

**Affiliations:** 1 Blood Cell Development and Function Program, Fox Chase Cancer Center, Philadelphia, PA 19111 USA; 2 Division of Inflammation Biology, La Jolla Institute for Allergy and Immunology, La Jolla, CA 92037 USA

## Abstract

Myeloid cells, key players in atherosclerosis, take up and present antigens, leading to systemic and local T cell activation. The recruitment and activation of immune cells to the aorta in atherosclerosis is regulated by adhesion molecules, chemokines and cytokines. IL-27R is an immunoregulatory signaling nod in autoimmune and infectious pathologies. IL-27R was shown to suppress T cells activation in atherosclerosis, however it’s possible role in myeloid cell accumulation and activation is not understood. Here we demonstrate that *Apoe* ^−*/*−^ *Il27ra* ^−*/*−^ mice fed with “Western Diet” for 7 or 18 weeks developed significantly more atherosclerosis compared to *Apoe* ^−*/*−^ *Il27ra* ^+*/*−^ controls. Accelerated disease was driven by enhanced expression of adhesion molecules and chemokines causing the accumulation of immune cells. Myeloid cells produced more inflammatory cytokines and upregulated MHCII. Multiphoton microscopy revealed more efficient interactions between aortic myeloid cells and CD4^+^ T cells. Overall, we show that IL-27R signaling controls endothelial cells activation and myeloid cell recruitment at early and advanced stages of atherosclerosis. In the absence of IL-27R myeloid cells become hyperactivated, produce pro-inflammatory cytokines and act as more potent antigen presenting cells. Enhanced interactions between *Il27ra* ^−*/*−^ APC and CD4^+^ T cells in the aortic wall contribute to T cells re-activation and pro-atherogenic cytokine production.

## Introduction

Atherosclerosis is a lipid-driven chronic inflammatory disease characterized by progressive atherosclerotic plaque growth accompanied by the accumulation, local proliferation and activation of various immune cells in the vessel wall^1, 2^. Cells accumulated in atherosclerotic plaques and surrounding tissues produce various “mediators of inflammation” such as cytokines and chemokines, fueling local inflammation and promoting atherosclerosis^3–6^. Healthy aortas contain small number of macrophages, T cells and other immune cells^2, 4^. At early stages of atherosclerosis, endothelial cell activation facilitates the initial phase of monocytes, neutrophils and T cells recruitment, and subsequent increase in production of chemokines and cytokines in the plaque results in further accumulation of inflammatory cells.

A key step in the immune response is the activation of T cells by antigens presented by antigen-presenting cells (APC). In a variety of inflammatory contexts, including atherosclerosis, such interactions take place in the lymph nodes^7, 8^. Activated T cells migrate to the site of inflammation, where they carry out their effector functions. However, local interaction of antigen-experienced CD4^+^ T cells with APC in the tissue was also reported to contribute to CD4^+^ T cells re-activation (recall response), which further fueled inflammatory response in various diseases, including atherosclerosis^9, 10^. In case of atherosclerosis, local abundance of lipoprotein-derived atherosclerotic antigens may constantly cause the activation of APC, antigen presentation and, thus, promote persistent local CD4^+^ T cells re-activation and as a consequence, upregulated cytokine production. However, mechanisms, which could negatively control this process, remain poorly understood.

Many cytokines have been implicated into pathogenesis of atherosclerosis^11, 12^, with only few of them, namely IL-10 and TGFβ, being athero-protective^13–16^. IL-27, a member of IL-6/IL-12 superfamily, is a critical regulator of immune responses. IL-27 cytokine is produced in response to inflammatory stimuli by myeloid cells and controls the activation and function of multiple hematopoietic cell subsets as well as some non-hematopoietic cells expressing IL-27 receptor (IL-27R)^17–19^. IL-27R is heterodimer of a unique IL-27ra and common gp130 chains. IL-27 consists of 2 subunits, p28 and Ebi3^20^. The heterodimeric structure of IL-27 protein complicates the investigation of this cytokine function using inactivation of genes encoding IL-27 subunits, because each of its subunits on its own also participates in the formation of other cytokines. Ebi3 heterodimerizes with IL-12p35 to form IL-35, another anti-inflammatory cytokine^21^, while the p28 subunit potentially forms a homodimer^22^ or binds with cytokine-like factor 1(CLF) to produce p28/CLF, a complex that engages IL-6R^23^. Because of such complexity, IL-27R deficient mice (lacking *Il27ra* gene) represent the best tool to address the role of IL-27R signaling in various physiological settings.

IL-27R signaling was shown to negatively control Th17 activation and IL-17A production in models of infection and autoimmunity^24, 25^. It was also shown to modulate Th1 cells activation and IFNγ production^26^. T regulatory (Treg) cells survival and functions are also dependent on IL-27R signaling, however both suppressive and activating role of IL-27R signaling on Tregs were reported by different groups^27, 28^, perhaps reflecting differences in mouse models used in the studies. IL-27R expression was also reported on myeloid cells^29^. *In vitro* studies demonstrated that IL-27 promotes inflammatory gene expression in myeloid cells, however, *in vivo* data suggest suppressive role of IL-27R signaling as determined by the enhanced MHCII expression on dendritic cells (DC) isolated from *Il27ra* ^−*/*−^ mice^30^.

IL-27R signaling was shown to limit atherosclerosis in *Ldlr* ^−*/*−^ atherosclerosis-prone mice with global^31^ or hematopoietic IL-27R deficiency^19^. Hematopoietic ablation of *Il27ra* accelerated atherosclerosis due to enhanced activation of CD4^+^ T cells, in particular, Th17 cells, accompanied by increased IL-17A, TNF-α and IL-6 production, CCL2 chemokine expression and accumulation of myeloid cells^19^. In addition to its ability to regulate cells of adaptive immunity (i.e. lymphocytes), IL-27R signaling can also potentially control atherosclerosis via regulation of innate immune cells, particularly macrophages and APC. Indeed, IL-27R signaling was shown to suppress macrophage activation and foam cell formation as determined by the analysis of peritoneal macrophage function in *Il27ra* ^−*/*−^ *Ldlr* ^−*/*−^ or bone marrow transplanted mice^31^.

The present study was designed to examine whether IL-27R signaling regulates antigen presentation in atherosclerosis. Here we elucidated the role of IL-27R in the *Apoe* ^−*/*−^ model of atherosclerosis, which shares similarities with, but also has important differences from the *Ldlr* ^−*/*−^ model. We assessed the role of IL-27R in early and advanced stages of the disease. Finally, we determined the role of IL-27R in antigen presentation in the aortic wall. Our data show that IL-27R signaling is critical in limiting both early and late stages of atherosclerosis by controlling myeloid cell accumulation (via regulation of adhesion molecule and chemokine expression), and regulating myeloid cells activation and antigen presentation (by limiting MHCII expression and cytokine production) in the aortas, subsequently affecting CD4^+^ T cells activation and atherosclerosis progression.

## Results

### IL-27R signaling suppresses atherosclerosis development in *Apoe*^−*/*−^ mice

To date a limited number of publications had tried to address the role of IL-27 in atherosclerosis, using *Ldlr* ^−*/*−^ model and bone marrow transplantation approach^19, 31^. Here we decided to test the role of IL-27R signaling in atherosclerosis progression in another atherosclerotic model- *Apoe* ^−*/*−^ mice and further characterize molecular and cellular mechanisms underlying IL-27R action. We crossed *Il27ra* ^−*/*−^ mice to *Apoe* ^−*/*−^ background to obtain *Apoe* ^−*/*−^ *Il27ra* ^−*/*−^ and *Apoe* ^−*/*−^ *Il27ra* ^+*/*−^ mice. Mice were fertile and healthy with no evidence for runting. It is known that even small differences in genetic background^32^ or microbiota^33, 34^ influences atherosclerosis and other inflammation-driven diseases. Therefore, we compared atherosclerosis progression in *Apoe* ^−*/*−^ *Il27ra* ^−*/*−^ and *Apoe* ^−*/*−^ *Il27ra* ^+*/*−^ cage mate and littermate controls, derived from the same parents and housed in the same cages through the entire duration of the experiment, to minimize these potentially confounding factors. Starting at 8 weeks after birth, these mice were fed with high fat “Western Diet” (WD) for 7 (“early” atherosclerosis) or 18 weeks (“advanced” atherosclerosis) to assess inflammatory changes in the aortic wall and atherosclerosis development at early and advanced stages of the model, respectively.

**Figure 1:**
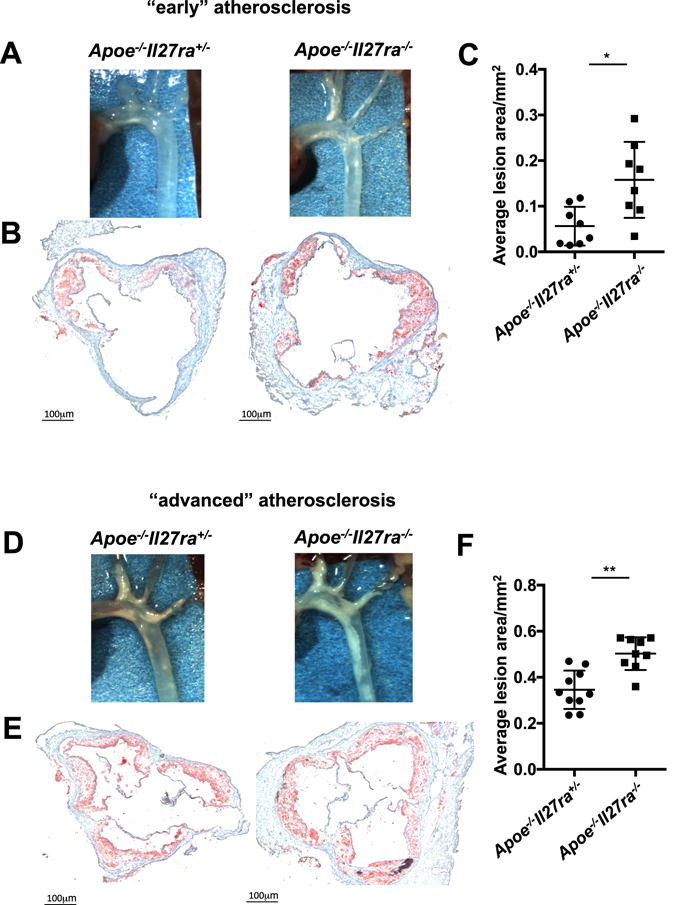
Accelerated atherosclerotic lesions development in *Apoe* ^−*/*−^ *Il27ra* ^−*/*−^mice. *Apoe* ^−*/*−^ *Il27ra* ^+*/*−^ or *Apoe* ^−*/*−^ *Il27ra* ^−*/*−^ mice were fed with Western diet (WD) for 7 (**A** **–C**) or 18 weeks (**D–F**). Atherosclerotic lesions in aortic arch (**A**) and aortic root sections (**B**) of *Apoe* ^−*/*−^ *Il27ra* ^+*/*−^ and *Apoe* ^−*/*−^ *Il27ra* ^−*/*−^ mice fed with WD for 7 weeks. (**C**) Quantitative comparison of aortic lesion size in *Apoe* ^−*/*−^ *Il27ra* ^+*/*−^ (n = 8) and *Apoe* ^−*/*−^ *Il27ra* ^−*/*−^ (n = 8) mice feeding with WD for 7 weeks. Atherosclerotic lesions in aortic arch (**D**) and aortic root sections of (**E**) *Apoe* ^−*/*−^ *Il27ra* ^+*/*−^ and *Apoe* ^−*/*−^ *Il27ra* ^−*/*−^ mice fed with WD for 18 weeks. (**F**) Quantitative comparison of aortic lesion size in *Apoe* ^−*/*−^ *Il27ra* ^+*/*−^ (n = 10) and *Apoe* ^−*/*−^ *Il27ra* ^−*/*−^ (n = 9) mice feeding with WD for 18 weeks. Data are mean ± SEM from 4 independent experiments.

Macroscopic and histological analyses revealed accelerated atherosclerosis progression in *Apoe* ^−*/*−^ *Il27ra* ^−*/*−^ mice already after 7 weeks of WD feeding compared to *Apoe* ^−*/*−^ *Il27ra* ^+*/*−^ cage mate and littermate controls (Fig. 1A,B). Quantitative analysis of atherosclerotic lesion area also revealed enhanced lesion size in roots of *Apoe* ^−*/*−^ *Il27ra* ^−*/*−^ mice fed with WD for 7 weeks compared to controls (Fig. 1C). Lipid profile and weight of mice from both cohorts remained unchanged (Supplementary Fig. S1A–C), indicating that effects of IL-27R deficiency on atherosclerosis are not directly mediated by global alterations in lipid homeostasis driven by the absence of IL-27R signaling. The analysis of blood leukocyte count revealed a significant reduction of circulating monocytes in *Apoe* ^−*/*−^ *Il27ra* ^−*/*−^ mice fed with WD for 7 weeks compared to controls, suggesting possible increased recruitment of these cells into the aorta (Supplementary Fig. S1D). No significant difference in number of hematopoietic bone marrow precursors was found between cohorts (data not shown).

Moreover, *Apoe* ^−*/*−^ *Il27ra* ^−*/*−^ mice also had significantly accelerated atherosclerosis progression at advanced stages of the disease, when fed with WD for 18 weeks (Fig. 1D,E). Atherosclerotic plaques in aortic roots of *Apoe* ^−*/*−^ *Il27ra* ^−*/*−^ mice were significantly enlarged compared to controls (Fig. 1F).

Taken together, our data demonstrate strong acceleration of atherosclerosis in an *Apoe* ^−*/*−^ mouse model at early and advanced stages of the disease in the absence of IL-27R signaling and for the first time specifically address the possible role of IL-27R during early stages of plaque development.

### Increased expression of chemokines and adhesion molecules in *Apoe*^−*/*−^*Il27ra*^−*/*−^ mice

**Figure 2:**
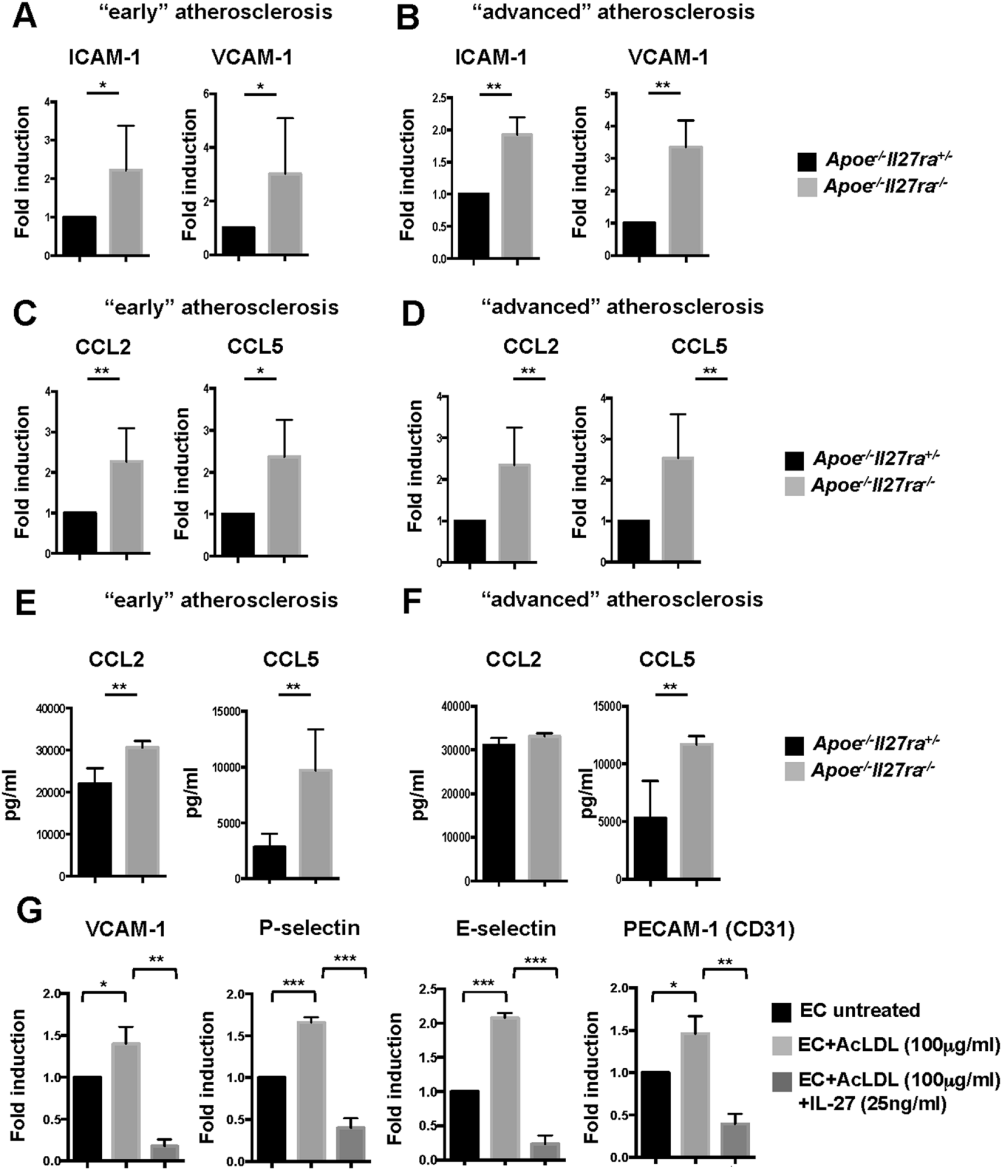
Upregulated expression of chemokines and adhesion molecules in aortas of *Apoe* ^−*/*−^ *Il27ra* ^−*/*−^mice. (**A**,**B**) Relative gene expression of adhesion molecules ICAM-1 and VCAM-1 in aortas of *Apoe* ^−*/*−^ *Il27ra* ^−*/*−^ (n = 5) mice fed with WD for 7 weeks (**A**) or 18 weeks (**B**) were normalized to L-32 and fold induction was calculated based on the gene expression in aortas of control *Apoe* ^−*/*−^ *Il27ra* ^+*/*−^ (n = 5) mice. (**C**,**D**) Relative gene expression of CCL2 and CCL5 in aortas of *Apoe* ^−*/*−^ *Il27ra* ^−*/*−^ (n = 5) mice fed with WD for 7 weeks (**C**) or 18 weeks (**D**) were normalized to L-32 gene expression and fold induction was calculated based on the gene expression in aortas of control *Apoe* ^−*/*−^ *Il27ra* ^+*/*−^ (n = 5) mice. (**E**,**F**) CCL2 and CCL5 were measured by multiplex cytokines array in supernatants obtained from aortic cell suspensions of *Apoe* ^−*/*−^ *Il27ra* ^+*/*−^ (n = 5) *or Apoe* ^−*/*−^ *Il27ra* ^−*/*−^ (n = 5) mice fed with WD for 7 weeks (**E**) or 18 weeks (**F**) stimulated with anti-CD3/anti-CD28 for 48 hours. (**G**) Relative gene expression of vascular adhesion molecule VCAM-1, P-selectin, E-selectin and PECAM-1 in endothelial cells (mLEC cell line) treated with acLDL (100 μg/ml) and rIL-27 (25ng/ml) were normalized to L-32 and fold induction was calculated based on the gene expression in untreated endothelial cells. Data are mean ± SEM from at least 2 independent experiments.

Early atherosclerosis is characterized by endothelial cell activation, increased expression of adhesion molecules and chemokines, mediating the recruitment of inflammatory cells to the aortic wall^3^. We sought to examine if IL-27R signaling regulates adhesion molecules expression at early stages of the disease. RT-qPCR analysis revealed strong induction of the adhesion molecule ICAM-1 and VCAM-1 expression in aortas of *Apoe* ^−*/*−^ *Il27ra* ^−*/*−^ mice fed with WD for 7 weeks compared to controls (Fig. 2A).

Various chemokines including CCL2 and CCL5 were shown to control the recruitment of myeloid cells to the aortas during atherosclerosis progression^35^. We examined chemokine expression in mice fed with WD for 7 weeks and found enhanced production of CCL2 and CCL5 chemokines in aortas of *Apoe* ^−*/*−^ *Il27ra* ^−*/*−^ mice compared to *Apoe* ^−*/*−^ *Il27ra* ^+*/*−^ controls (Fig. 2C,E). CCL2 and CCL5 chemokine productions were also enhanced in the spleen and paraaortic lymph node (paLN) of *Apoe* ^−*/*−^ *Il27ra* ^−*/*−^ mice (Supplementary Fig. S2A). Similar to the early stages of atherosclerosis, advanced atherosclerotic lesions were also characterized by elevated expression of adhesion molecules (Fig. 2B) and chemokines (Fig. 2D,F) in aortas, spleen and paLN (Supplementary Fig. S2B) of *Apoe* ^−*/*−^ *Il27ra* ^−*/*−^ mice fed with WD for 18 weeks.

Because vascular endothelial cells can be activated in the inflammatory environment *in vivo* by multiple stimuli including modified low-density lipoproteins, we decided to examine if IL-27 has direct effect on cultured endothelial cells in the presence of acetylated LDL (AcLDL). We pretreated stable cell line of lung endothelial cells (mLEC) with acLDL (100 μg/ml) for 6 hours followed by stimulation with rIL-27 (25ng/ml) and assessed changes in gene expression 24 h later. AcLDL activated the expression of several adhesion molecules including VCAM-1, P-selectin, E-selectin and PECAM-1, while this effect was strongly diminished in the presence of recombinant IL-27 (Fig. 2G). Taken together, these data suggest that IL-27 has a direct effect on endothelial cells preventing the excessive expression of potentially pro-inflammatory adhesion molecules.

Thus, our data suggest that during atherosclerosis development and progression IL-27R signaling may regulate endothelial cells function and drives the suppression of adhesion molecule and chemokine expression, thereby preventing excessive accumulation of immune cells.

### IL-27R deficiency accelerates immune cell accumulation in the aorta

**Figure 3:**
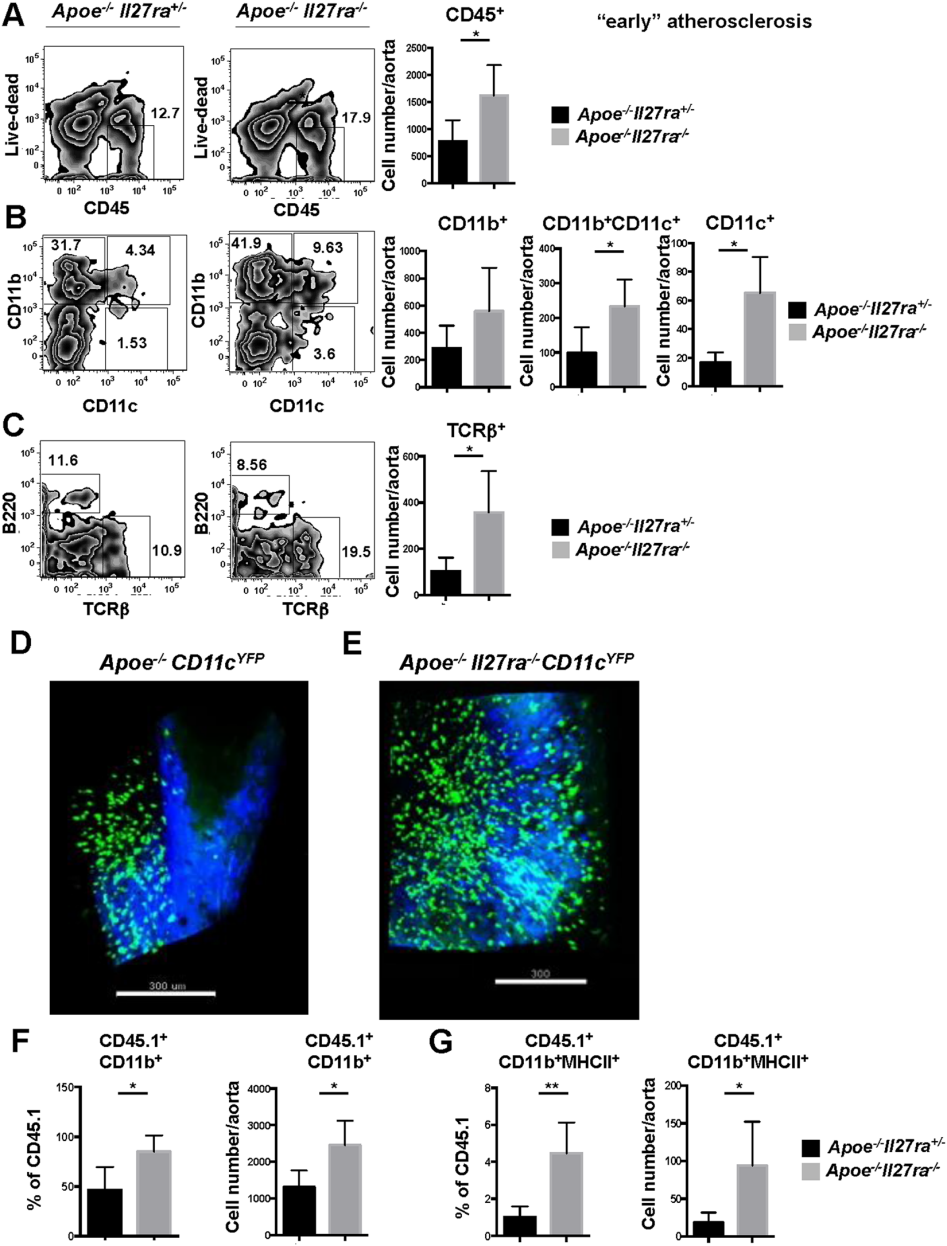
Enhanced accumulation of immune cells in aortas of *Apoe* ^−*/*−^ *Il27ra* ^−*/*−^ mice. Live CD45^+^ cells from aortas of *Apoe* ^−*/*−^ *Il27ra* ^+*/*−^ or *Apoe* ^−*/*−^ *Il27ra* ^−*/*−^ mice fed with WD for 7 weeks were stained for CD45^+^, CD11b^+^, CD11c^+^ and TCRβ^+^. Percentage (**left**) and cell number (**right**) (**A–C**) of live CD45^+^, CD11b^+^CD11c^−^, CD11b^+^CD11c^+^ and CD11b- CD11c^+^ cells, TCRβ^+^ T cells in aortas of *Apoe* ^−*/*−^ *Il27ra* ^+*/*−^ or *Apoe* ^−*/*−^ *Il27ra* ^−*/*−^ mice fed with WD for 7 weeks was quantified by flow cytometry. Data are mean ± SEM from at least 3 independent experiments. Accumulation of CD11c^YFP+^ APC in aortas of *Apoe* ^−*/*−^ (**D**) and *Apoe* ^−*/*−^ *Il27ra* ^−*/*−^ (**E**) mice was analyzed by 2 photon microscopy. Green – CD11c^YFP+^ APC cells, blue – collagen detected by second harmonics generation. CD45.1^+^ monocytes from B6 mice were adoptively transferred to *Apoe* ^−*/*−^ *Il27ra* ^+*/*−^ (n = 5) or *Apoe* ^−*/*−^ *Il27ra* ^−*/*−^ (n = 6) mice fed with WD for 7 weeks. Monocyte recruitment to the aortas was assessed by flow cytometry 48 hours after cell transfer. Percentage and absolute number of recruited monocytes (**F**) and MHCII expression by recruited CD45.1 CD11b^+^ monocytes (**G**) in the aortic wall of *Apoe* ^−*/*−^ *Il27ra* ^−*/*−^ and *Apoe* ^−*/*−^ *Il27ra* ^+*/*−^ mice. Data are mean ± SEM from 2 independent experiments.

One key signature underlying atherosclerosis progression is the accumulation of various immune and inflammatory cells in the vessel wall both in the plaque area and surrounding adventitia^36^. We performed flow cytometry analysis and assayed the composition of immune cells in aortas of *Apoe* ^−*/*−^ *Il27ra* ^−*/*−^ and *Apoe* ^−*/*−^ *Il27ra* ^+*/*−^ control mice. We found increased accumulation of CD45^+^ leukocytes (Fig. 3A): including CD11b^+^CD11c^−^, CD11b^+^CD11c^+^ and CD11b^−^CD11c^+^ myeloid cell subsets (Fig. 3B) in aortas of *Apoe* ^−*/*−^ *Il27ra* ^−*/*−^ mice fed with WD for 7 weeks compared to *Apoe* ^−*/*−^ *Il27ra* ^+*/*−^ controls. Moreover, we also observed increased accumulation of T cells in aortas of IL-27R deficient mice (Fig. 3C). Further accumulation of immune cells subsets was also found in mice fed with WD for 18 weeks (Supplementary Fig. S3A–C).

Whole mount imaging of aortas to visualize CD11c^YFP+^ cells (Fig. 3D,E and Supplementary movies S1 and 2) and immunofluorescent staining demonstrated the localization and increased accumulation of myeloid cells in aortic roots of *Apoe* ^−*/*−^ *Il27ra* ^−*/*−^ mice compared to *Apoe* ^−*/*−^ *Il27ra* ^+*/*−^ controls (Supplementary Fig. S3D).

To determine if increased myeloid cells accumulation is due to enhanced recruitment of monocytes, we adoptively transferred CD45.1^+^ monocytes isolated from B6/CD45.1 congenic mice into *Apoe* ^−*/*−^ *Il27ra* ^−*/*−^ or *Apoe* ^−*/*−^ *Il27ra* ^+*/*−^ controls fed with WD for 7 weeks. Flow cytometry analysis revealed increased percentage and absolute number of recruited monocytes in the aortic wall of *Apoe* ^−*/*−^ *Il27ra* ^−*/*−^ mice 48 h after monocyte transfer (Fig. 3F). Recruited monocytes were characterized by elevated MHCII expression when transferred into *Apoe* ^−*/*−^ *Il27ra* ^−*/*−^ hosts (Fig. 3G). These results suggest that myeloid cells accumulation in aortas of *Apoe* ^−*/*−^ *Il27ra* ^−*/*−^ can be at least partially due to increased monocyte recruitment.

### Enhanced activation of myeloid and T cells in *Apoe*^−*/*−^*Il27ra*^−*/*−^ mice

**Figure 4:**
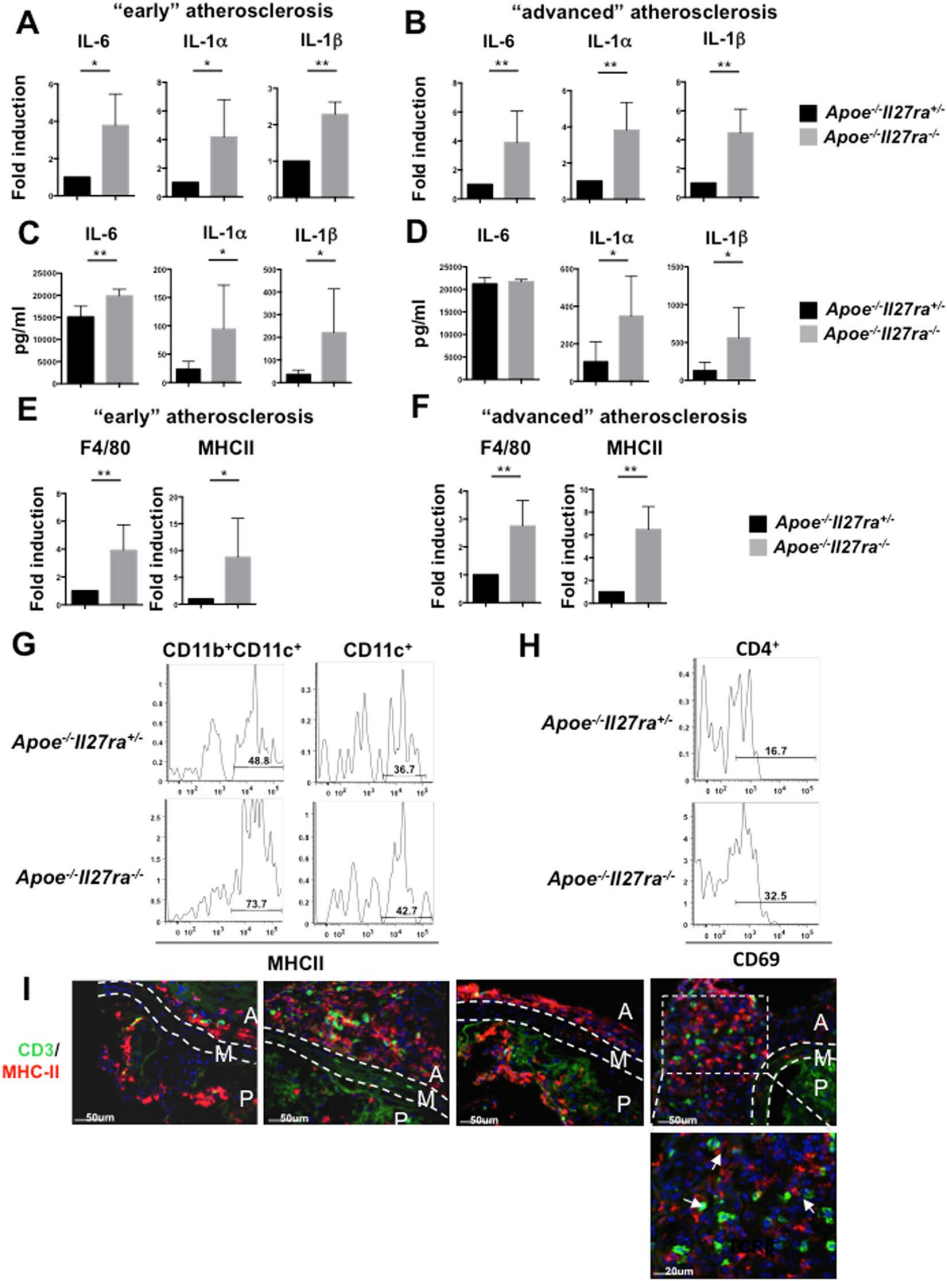
Enhanced activation of myeloid cells and T cells in *Apoe* ^−*/*−^ *Il27ra* ^−*/*−^ mice. (**A**,**B**) Relative gene expression of IL-6, IL-1α and IL-1β in aortas of *Apoe* ^−*/*−^ *Il27ra* ^−*/*−^ (n = 5) mice fed with WD for 7 weeks (**A**) or 18 weeks (**B**) were normalized to L-32 gene expression and then normalized to gene expression in aortas of control *Apoe* ^−*/*−^ *Il27ra* ^+*/*−^ (n = 5) mice. (**C**,**D**) IL-6, IL-1α and IL-1β were measured by bead array in supernatants of aortic cell suspension obtained from *Apoe* ^−*/*−^ *Il27ra* ^+*/*−^ (n = 5) or *Apoe* ^−*/*−^ *Il27ra* ^−*/*−^ (n = 5) mice fed with WD for 7 weeks (**C**) or 18 weeks (**D**), stimulated with anti-CD3/anti-CD28 for 48 hours. (**E**,**F**) Relative gene expression of F4/80 and MHCII in aortas of *Apoe* ^−*/*−^ *Il27ra* ^−*/*−^ (n = 5) mice fed with WD for 7 weeks (**E**) or 18 weeks (**F**) were normalized to L-32 gene expression and fold induction was calculated based on the gene expression in aortas of control *Apoe* ^−*/*−^ *Il27ra* ^+*/*−^ (n = 5) mice. (**G**) Expression of MHCII by CD11b^+^CD11c^+^ and CD11c^+^ cells in aorta of *Apoe* ^−*/*−^ *Il27ra* ^+*/*−^ or *Apoe* ^−*/*−^ *Il27ra* ^−*/*−^ mice fed with WD for 7 weeks. (**H**) Expression of CD69, a marker of T cell activation, by CD4^+^ T cells in aortas of *Apoe* ^−*/*−^ *Il27ra* ^+*/*−^ or *Apoe* ^−*/*−^ *Il27ra* ^−*/*−^ mice fed with WD for 18 weeks. Data are mean ± SEM from 3 independent experiments. (**I**) Localization of CD3^+^ T cells and MHCII^+^ cells in aortic roots of *Apoe* ^−*/*−^ *Il27ra* ^+*/*−^ or *Apoe* ^−*/*−^ *Il27ra* ^−*/*−^ mice fed with WD for 7 weeks (early lesions) or 18 weeks (advanced lesions) as demonstrated by confocal imaging. Arrows show co-localization of CD3^+^ T cells and MHCII^+^ cells.

To gain insights into functional role and activation perturbations of immune cells accumulated in atherosclerotic aortas, we first analyzed cytokines in supernatants obtained from the aortas of *Apoe* ^−*/*−^ *Il27ra* ^−*/*−^ mice and controls fed with WD for 7 weeks or 18 weeks. We found increased production of myeloid cell-derived cytokines, including IL-6, IL-1α and IL-1β (Fig. 4A–D) in aortas obtained from *Apoe* ^−*/*−^ *Il27ra* ^−*/*−^ mice. These data suggest that IL-27R signaling not only controls the recruitment of inflammatory cells to the aortic wall, but also regulates their activation and myeloid cell-derived pro-inflammatory cytokine production.

To assess the effect of IL-27R deficiency on antigen presentation, we measured MHCII gene expression by RT-qPCR and its surface protein expression by flow cytometry. This analysis revealed enhanced MHCII expression on CD11b^+^CD11c^+^ and CD11b^−^CD11c^+^ APC in the aorta (Fig. 4E–G) and spleen as well as in paLN (Supplementary Fig. S4A,B) of *Apoe* ^−*/*−^ *Il27ra* ^−*/*−^ mice.

Upregulation of MHCII surface expression indicates more profound maturation and activation of APC and enhanced antigen presentation, which in turn should promote T cells activation. Indeed, we found elevated expression of CD69 on T cells in aortas (Fig. 4H) and spleens (Supplementary Fig. S4C) of *Apoe* ^−/−^ *Il27ra* ^−*/*−^ mice, suggesting enhanced CD4^+^ T cells activation in these mice. Immunofluorescent staining revealed also increased co-localization of T cells with MHCII expressing cells in aortic roots of *Apoe* ^−*/*−^ *Il27ra* ^−*/*−^ mice compared to controls (Fig. 4I). Taken together, our data show that in atherosclerosis, IL-27R signaling controls myeloid cell activation in early and advanced atherosclerosis and suggest that in the absence of competent IL-27R signaling, excessive myeloid cell activation may contribute to heightened T cell responses.

### IL-27R deficiency enhances local antigen presentation

Atherosclerosis progression in part is modulated by local interactions between APC and CD4^+^ T cells in the aortic wall^9^. Such interactions serve to promote local re-activation of CD4^+^ T cells, subsequent production of inflammatory pro-atherogenic cytokines^9^.

To determine if IL-27R signaling regulates local antigen presentation in the aortas, we employed 2 photon microscopy to visualize and characterize interactions between APC and CD4^+^ T cells. To assay and image antigen presentation in aortas of atherosclerotic mice, we bred composite *Apoe* ^−*/*−^ *Il27ra* ^−*/*−^CD11c^YFP^ double knockout-transgenic mice and compared them to *Apoe* ^−*/*−^CD11c^YFP^ controls. While various subsets of dendritic cells, monocytes and macrophages are CD11c^YFP+^ using flow cytometry based detection, we previously demonstrated that in CD11c^YFP^ mice, CD11b^+^CD11c^+^ myeloid cells are primarily labeled with Yellow fluorescent protein (YFP) at fluorescence intensities sufficient for detection by 2 photon microscopy^9^.

**Figure 5:**
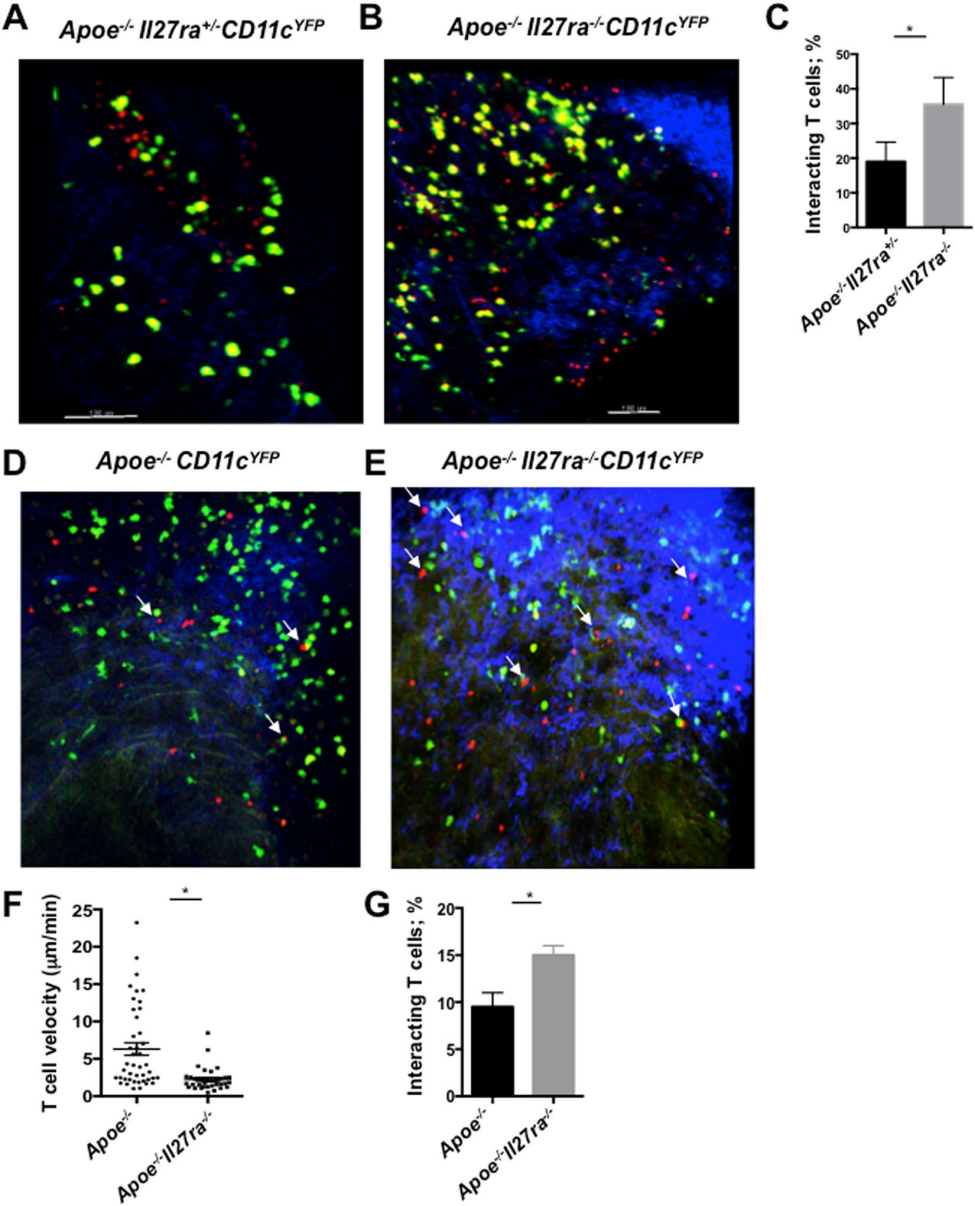
IL-27R deficiency promotes CD4^+^ T cells-APC interactions in the aortas. CD4^+^ T cells were labeled by administration of anti-CD4-PE antibody into live *Apoe* ^−*/*−^ and *Apoe* ^−*/*−^ *Il27ra* ^−*/*−^ mice after 5 weeks of WD feeding and localization of CD11c^YFP+^ APC and CD4-PE^+^ T cells were imaged by 2 photon and confocal microscopy in aortas of *Apoe* ^−*/*−^ *Il27ra* ^+*/*−^ *CD11c* ^*YFP*^ (**A**) and *Apoe* ^−*/*−^ *Il27ra* ^−*/*−^ *CD11c* ^*YFP*^ (**B**) mice. (**C**) Quantification of co-localizing cells in *Apoe* ^−*/*−^ *Il27ra* ^+*/*−^ *CD11c* ^*YFP*^ and *Apoe* ^−*/*−^ *Il27ra* ^−*/*−^ *CD11c* ^*YFP*^ aortas. (**D**,**E**) Two photon microscopy imaging revealed increased number of interacting cells in explanted aortas of *Apoe* ^−*/*−^ *Il27ra* ^−*/*−^ *CD11c* ^*YFP*^ mice (**E**) compared to control *Apoe* ^−*/*−^ *CD11c* ^*YFP*^ (**D**) fed with WD for 15 weeks. Isolated aortas were co-cultured with sorted from spleens CD4^+^ T cells obtained from atherosclerotic *Apoe* ^−*/*−^ *Il27ra* ^−*/*−^ mice. (**F**) Percent of CD4^+^ T cells interacting with CD11c^YFP+^ APC. (**G**) Reduced velocity of CD4^+^ T cells in *Apoe* ^−*/*−^ *Il27ra* ^−*/*−^ *CD11c* ^*YFP*^ aortas compared to *Apoe* ^−*/*−^ *CD11c* ^*YFP*^ aortas. Data are mean ± SEM from 4 independent experiments.

First, we assayed endogenous localization of APC and CD4^+^ T cells in early atherosclerotic lesions. We administered anti-CD4 PE antibody into live *Apoe* ^−*/*−^ *Il27ra* ^+*/*−^ CD11c^YFP^ or *Apoe* ^−*/*−^ *Il27ra* ^−*/*−^ CD11c^YFP^ mice to label endogenous CD4^+^ T cells. Aortas were isolated 30 min after antibody administration and the localization of APC (labeled by CD11c^YFP^) or CD4^+^ T cells (labeled by PE) was assessed by 2 photon microscopy. In agreement with our flow cytometry data, we found higher numbers of both YFP^+^ cells and CD4-PE^+^ cells in aortas of *Apoe* ^−*/*−^ *Il27ra* ^−*/*−^ mice (Fig. 5A,B). We also found higher number APC and CD4^+^ T cells, which co-localized with each other, implying their possible local interactions in the atherosclerotic tissue (Fig. 5C).

To assay the role of IL-27R signaling in regulation of antigen presentation, we utilized a method of explanted aorta live imaging, which we have previously developed^9^. We sorted CD4^+^ T cells from the spleens and paLNs of *Apoe* ^−*/*−^ *Il27ra* ^−*/*−^ mice fed with WD for 16 weeks and labeled them with SNARF dye. These labeled CD4^+^ T cells derived from atherosclerotic mice were co-cultured with explanted aortas from *Apoe* ^−*/*−^ *Il27ra* ^−*/*−^CD11c^YFP^ and *Apoe* ^−*/*−^CD11c^YFP^ mice. The behavior and APC-CD4^+^ T cells interactions were imaged by 2 photon microscopy 12 h later. In agreement with our observations of increased MHCII expression (Fig. 4E–G) and increased numbers of co-localizing APC and CD4^+^ T cells (Figs 4I and 5A,B), we found a higher percentage of APC-CD4^+^ T cells interactions in *Apoe* ^−*/*−^ *Il27ra* ^−*/*−^ CD11c^YFP^ aortas compared to *Apoe* ^−*/*−^CD11c^YFP^ controls (Fig. 5D–F and Supplementary movies S3 and 4). Our earlier work revealed that only activated memory T cells are capable of migrating to aortas and interacting there with APC. We also previously proved that MHCII blockade completely abrogate CD4^+^ T cells-APC interactions, underlying the importance of MHCII expression in local intra-aortic APC- T cells interactions^9^.

One important characteristic of the productive antigen presentation is the reduction of CD4^+^ T cells velocity and their prolonged co-localization with APC^8, 9^. Indeed, we found a reduction of CD4^+^ T cells speed (Fig. 5F) as well as higher percentage of APC-CD4^+^ T cells interactions in *Apoe* ^−*/*−^ *Il27ra* ^−*/*−^ aortas (Fig. 5G).

Overall our data demonstrate a suppressive role of IL-27R signaling on myeloid cells activation, antigen presentation and interaction with CD4^+^ T cells, thus positioning IL-27R signaling as an important regulator of immune response in atherosclerosis.

### Elevated expression of pro-inflammatory cytokines in IL-27R deficient mice

**Figure 6:**
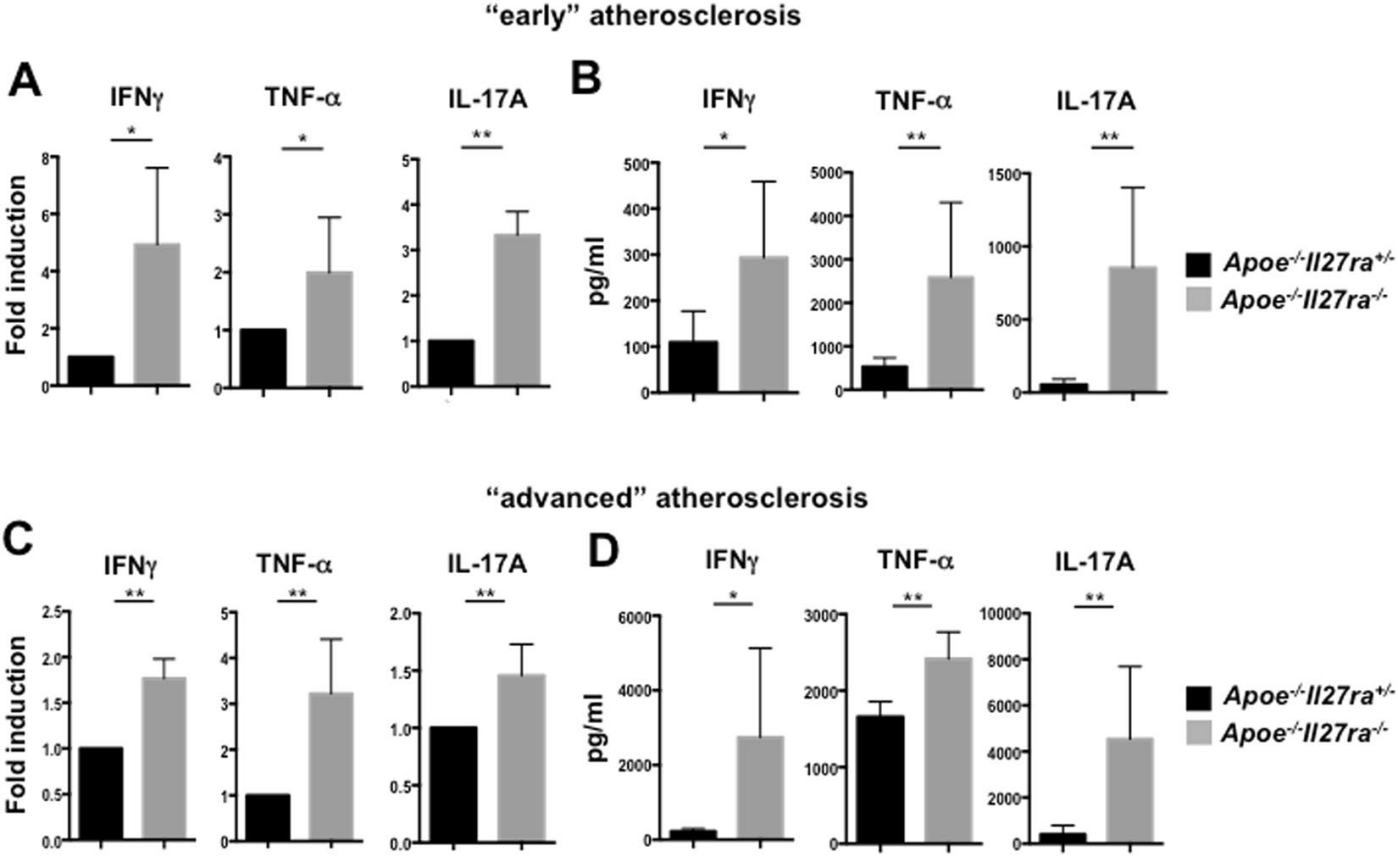
Increased expression of pro-inflammatory cytokines in aortas of *Apoe* ^−*/*−^ *Il27ra* ^−*/*−^ mice. (**A**,**C**) Relative gene expression of IFNγ, TNF-α and IL-17A in aortas of *Apoe* ^−*/*−^ *Il27ra* ^−*/*−^ (n = 5) mice fed with WD for 7 weeks (**A**) or 18 weeks (**C**) were normalized to L-32 gene expression and fold induction was calculated based on the gene expression in aortas of control *Apoe* ^−*/*−^ *Il27ra* ^+*/*−^ (n = 5). (**B**,**D**) Production of pro-inflammatory cytokines IFNγ, TNF-α and IL-17A in aortas of *Apoe* ^−*/*−^ *Il27ra* ^+*/*−^ or *Apoe* ^−*/*−^ *Il27ra* ^−*/*−^ mice at different stages of atherosclerosis was measured by multiplex cytokines array in supernatants of aortic cell suspension obtained from *Apoe* ^−*/*−^ *Il27ra* ^+*/*−^ (n = 5) or *Apoe* ^−*/*−^ *Il27ra* ^−*/*−^ (n = 5) mice fed with WD for 7 (**B**) or 18 weeks (**D**), stimulated with anti-CD3/anti-CD28 for 48 hours. Data are mean ± SEM from 3 independent experiments.

Local T cell re-activation promotes T cell-derived cytokine production^9^. We compared cytokines produced by T cells in aortas of *Apoe* ^−*/*−^ *Il27ra* ^−*/*−^ and *Apoe* ^−*/*−^ *Il27ra* ^+*/*−^ mice with early and advanced atherosclerotic lesions. We found upregulated production of several pro-inflammatory cytokines including IFNγ, TNF-α, IL-17A in aortas of *Apoe* ^−*/*−^ *Il27ra* ^−*/*−^ mice compared to *Apoe* ^−*/*−^ *Il27ra* ^+*/*−^ control mice with early lesions (Fig. 6A,B). The production of these cytokines was further elevated in aortas with advanced lesions (Fig. 6C,D). Similar changes of pro-inflammatory cytokine production were also found in the spleen and paLN both at early and advanced stages of the disease (Supplementary Fig. S5).

These data demonstrate enhanced T cell derived cytokine production, elevated at least partially due to enhanced interactions with APC. IL-27R signaling, therefore, was found to have an essential immunoregulatory role, reducing expression of myeloid- (Fig. 4A–D) and T cell-derived cytokines (Fig. 6) both in early and in advanced stages of atherosclerosis. Some of these pro-inflammatory cytokines are also known regulators of chemokines, which in turn drive immune cell recruitment, providing positive feedback loop in atherosclerosis progression.

Taken together, our data extend the previously suggested anti-inflammatory role of IL-27R signaling in atherosclerosis to the *Apoe* ^−*/*−^ model, demonstrate its important contribution both at early and advanced stages of the disease and show a previously unrecognized mechanism of IL-27R signaling in regulating antigen presentation in the aortic wall.

## Discussion

The role of IL-27 in various models of infections was extensively investigated in the last decade^17, 18, 24, 25^. However, the role of IL-27/IL-27R signaling axis in chronic inflammatory diseases is still poorly understood. The mechanisms of immunoregulatory IL-27R signaling in atherosclerosis are largely unknown with only two publications attempted to address it in animal models.

It was suggested by previous work in *Ldlr* ^−*/*−^ model that IL-27R signaling serves as an important anti-inflammatory stimulus in atherosclerosis, as demonstrated in mice with global or hematopoietic IL-27R deficiency^19, 31^. IL-27R signaling was implicated in the regulation of foam cell formation since an enhanced oxLDL uptake was observed in macrophages obtained from *Ldlr* ^−*/*−^ atherosclerosis-prone IL-27R deficient mice^19, 31^. Moreover, IL-27R deficiency in hematopoietic cells led to accelerated disease progression in *Ldlr* ^−*/*−^ mice, in particular via upregulated IL-17A production by CD4^+^ T cells^19^, thereby illuminating an important role of IL-27R signaling in controlling of adaptive immunity in atherosclerosis.

Here we evaluated the role of IL-27R signaling in the vessel wall inflammation and assayed atherogenesis in another atherosclerosis model (*Apoe* ^−*/*−^ mice). We found that IL-27R signaling is required to suppress inflammation and atherosclerosis in *Apoe* ^−*/*−^ mice both at early (7 weeks of WD) and advanced (18 weeks of WD) stages of the disease. We showed that IL-27R signaling controls endothelial cells activation, chemokine production as well as suppresses myeloid cells and T cells accumulation and activation. Moreover, we found increased level of maturation and enhanced MHCII expression on APC from *Apoe* ^−*/*−^ *Il27ra* ^−*/*−^ mice, which led in enhanced antigen presentation and interaction with CD4^+^ T cells in the vessel wall as determined by 2 photon microscopy.

IL-27R is expressed by intestinal epithelial cells^37^ and aortic endothelial cells^19^, suggesting its potential role in regulation of these cell types. Activation of endothelial cells is an important step facilitating immune cells recruitment to the aortic wall in atherosclerosis. IFNγ, TNF-α and IL-4 cytokines were shown to regulate endothelial cells activation and adhesion molecules expression, therefore favoring the recruitment of specific subsets of effector T cells^38, 39^. Here we found that IL-27R signaling during the early phase of atherosclerosis development is implicated into the regulation of the adhesion molecules VCAM-1, P-selectin, E-selectin and PECAM-1 expression by vascular endothelial cells. We showed that IL-27R deficient *Apoe* ^−*/*−^ mice expressed significantly higher level of VCAM-1 and ICAM-1 in the aortas already at 7 weeks of WD feeding. Elevated level of adhesion molecules expression persists also at advanced stages of the disease suggesting continuous effect of IL-27R signaling on the suppression of endothelial cells activation in atherosclerosis. Even though previous study did not find any effect of recombinant IL-27 on VCAM-1 expression by HUVEC^31^, our data suggest that IL-27R signaling *in vivo* is important suppressor of endothelial cells activation. Moreover, our *in vitro* studies support our *in vivo* observations, since rIL-27 treatment significantly reduced the expression of several adhesion molecule genes including VCAM-1, P-selectin, E-selectin and PECAM-1 elevated in response to acLDL treatment of cultured endothelial cells. Possible discrepancies between two studies could be explained by variations in experimental system used: human venular endothelial cells^31^ or mouse lung endothelial cells, examined in our study. The analysis of chemokine production revealed upregulated CCL2 and CCL5 chemokines production in *Apoe* ^−*/*−^ *Il27ra* ^−*/*−^ mice compared to controls. Overall these data suggest an important role for IL-27R signaling in controlling cell recruitment in atherosclerosis.

Enhanced adhesion molecules expression and chemokine production in the absence of IL-27R signaling therefore conspire in increased accumulation of immune cells in the aortic wall. Similar to previously described in *Ldlr* ^−*/*−^ mice^19, 31^, here we also found that *Il27ra* ^−*/*−^ deficiency in *Apoe* ^−*/*−^ mice led to higher numbers of CD45^+^ cells recruited to the aorta, among them TCRβ^+^ T cells as well as several subsets of myeloid cells; namely CD11b^+^CD11c^−^, CD11b^+^CD11c^+^ and CD11b^−^CD11c^+^ cells.

Notably, here we showed that IL-27R deficient *Apoe* ^−*/*−^ mice were not only characterized by higher numbers, but also increased activation of myeloid cells subsets in the aortas. The expression of IL-27R on myeloid cells was previously reported, but its possible direct signaling role is still unclear. Previous studies demonstrated that stimulation of macrophages with recombinant IL-27 *in vitro* promotes pro-inflammatory gene expression^29^. However, *in vivo* the opposite was noted, i.e. IL-27R deficient DC were shown to be more activated and produced more pro-inflammatory cytokines upon LPS stimulation^30^. Similar findings were reported for peritoneal macrophages isolated from IL-27R deficient *Ldlr* ^−*/*−^ mice, where increased CCL2 and IL-6 production was detected^31^. Our data in *Apoe* ^−*/*−^ model of atherosclerosis argues for an important anti-inflammatory role of IL-27R signaling in myeloid cell compartment as demonstrated by the analysis of myeloid subsets composition and activation status in atherosclerotic *Apoe* ^−*/*−^ *Il27ra* ^−*/*−^ mice. The analysis of the cytokine spectrum produced in the aorta revealed increased levels of IL-1α, IL-1β and IL-6. These cytokines are produced primarily by myeloid cells in a variety of physiological settings^40^, including atherosclerosis^41^, and were previously shown to be pro-atherogenic^42–44^.

Most importantly, CD11b^+^CD11c^+^ and CD11b^−^CD11c^+^ myeloid cells from *Apoe* ^−*/*−^ *Il27ra* ^−*/*−^ mice exhibited significantly higher surface expression of MHCII both in aortas and secondary lymphoid organs, indicating their enhanced maturation. Importantly, an up-regulation of MHCII expression on myeloid cell subsets was detected already at 7 weeks of WD feeding, i.e. during early stage of the disease development.

Antigen presentation is a key step in activation of CD4^+^ T cells in inflammatory settings, including atherosclerosis^9, 45^. Typically antigen-presentation occurs in specialized lymphoid organs, however, local interactions and their role in the maintenance of T cells activation and local inflammation have been described^9, 10, 46^. We have previously demonstrated that antigen presenting cells can interact in the aortic wall with CD4^+^ T cells, resulting in their local re-activation and enhanced cytokine production^9^. Analysis of APC function in *Apoe* ^−*/*−^ *Il27ra* ^−*/*−^ mice revealed increased frequencies and longevities of interactions between CD4^+^ T cells and APC in aortas of *Apoe* ^−*/*−^ *Il27ra* ^−*/*−^ mice compared to controls at early and advanced stages of the disease, implicating heightened level of local antigen presentation. These data allow us to propose previously unexplored role of IL-27R signaling in regulation of APC function in atherosclerosis.

Previous work demonstrated that cytokines produced by re-activated T cells promote macrophage activation, scavenger receptor expression and foam cell formation, therefore promoting atherosclerosis^9^. Here, in *Apoe* ^−*/*−^ model, we detected elevated level of T cell-derived, pro-inflammatory cytokines including IL-17A, IFNγ and TNF-α in the aortas of *Apoe* ^−*/*−^ *Il27ra* ^−*/*−^ mice compared to *Apoe* ^−*/*−^ *Il27r* ^+*/*−^ controls. The elevated level of T cell derived cytokines indicates heightened activation of T cells, which is at least partially driven by their increasingly productive interactions with APC in the absence of regulatory IL-27R signaling.

Because of the broad IL-27R expression, most likely multiple mechanisms and cell subsets are contributing to the disease pathogenesis in an IL-27R dependent manner. Earlier well-documented studies demonstrated the direct role of IL-27R signaling on multiple T helper cell subsets^17^. Our previous work elucidated suppressive role of IL-27R signaling in the regulation of T cell function in atherosclerosis in *Ldlr* ^−*/*−^ mice with hematopoietic deficiency of IL-27R. Hirase at al. further demonstrated immunosuppressive role of IL-27 (Ebi3) and IL-27R signaling within the hematopoietic system and implicated IL-27R signaling into the regulation of foam cell formation^31^. Here we propose another mechanism, which can act in parallel with the already described mechanisms, by which IL-27R signaling directly controls inflammation within the vessel wall. Our data suggest that due to the immunoregulatory role of IL-27R in antigen presenting cell in the aorta, IL-27R therefore may indirectly suppress CD4^+^ T cells activation and production of pro-atherogenic T cell derived cytokines via controlling efficiency of antigen presentation.

Overall our data suggest that IL-27R signaling in atherosclerosis is an important regulator of both innate and adaptive immunity at early and late stages of the disease. Functional IL-27R signaling is involved in the regulation of endothelial cells activation and adhesion molecules expression. IL-27R signaling also controls myeloid cells activation, and myeloid cell-derived cytokine production and antigen presentation, which are essential for the induction and maintenance of T cell activation. This enhances our understanding of direct effects of IL-27R signaling on lymphocytes. Further studies using cell specific deletion of IL-27R will be required to address cell specific role of IL-27R cytokine signaling in inflammation in atherosclerosis.

## Materials and Methods

### Mice, Diet and cell lines

*Il27ra* ^−*/*−^ mice (JAX #018078) were crossed to *Apoe* ^−*/*−^ mice (JAX #002052) or to previously generated in the lab *Apoe* ^−*/*−^CD11c^YFP^ mice. *Apoe* ^−*/*−^ *Il27ra* ^+*/*−^CD11c^YFP^ mice were bred to *Apoe* ^−*/*−^ *Il27ra* ^+*/*−^CD11c^YFP^ to generate *Apoe* ^−*/*−^ *Il27ra* ^−*/*−^CD11c^YFP^, *Apoe* ^−*/*−^ *Il27r* ^+*/*−^CD11c^YFP^ and *Apoe* ^−*/*−^CD11c^YFP^ mice for imaging experiments. *Apoe* ^−*/*−^ *Il27ra* ^+*/*−^ were bred to *Apoe* ^−*/*−^ *Il27ra* ^−*/*−^ mice for all other studies.

CD45.1 (JAX #002014) mice were from Jackson Labs and bred in house. Mice were bred and housed under specific pathogen-free conditions in an AAALAC-approved barrier facility at Fox Chase Cancer Center (FCCC). The genotyping was performed by standard polymerase chain reaction protocols. Both male and female mice were used in the study. Animal numbers for each experiment are given in the Figure legends. *Apoe* ^−*/*−^ *Il27ra* ^+*/*−^ and *Apoe* ^−*/*−^ *Il27ra* ^−*/*−^ mice were fed with “Western Diet” (Teklad TD 88137) for 7 or 18 weeks beginning at 8 weeks after birth. Animal experiments were approved by the Animal Care Committee (IACUC) at FCCC.

Mice for live imaging experiments were bred and housed under specific pathogen-free conditions and fed with WD for various time points beginning at 8 weeks after birth in an AAALAC-approved barrier facility at La Jolla Institute for Allergy and Immunology (LJI). All imaging experiment procedures were approved by the Animal Care Committee at LJI.

Mouse lung endothelial cell line (mLEC) was kindly provided by Drs. Jonathan Chernoff and Maria Radu (FCCC). Cell were cultured in DMEM containing 20% FBS, supplemented with Endothelial Cell Growth Factor (75 μg/ml) (Sigma). To assess changes on gene expression cells were pretreated with acLDL (100 μg/ml) (Invitrogen) for 6 hours at 37 °C in serum free media, followed by for 24 h incubation with rIL-27 (25ng/ml) (eBioscience).

All methods were performed in accordance with the relevant institutional guidelines.

### Quantification of Aortic Atherosclerotic Lesions

To quantify aortic atherosclerosis lesions, aortic roots were isolated from the hearts and frozen on dry ice in O.C.T. (Optimal Cutting Temperature) compound Tissue Tek (Sakura) and stored at −80 °C. 5 μm sections were taken starting at the aortic valve plane and covering 400 μm in intervals of 50 μm. Sections were stained with Oil Red O/hematoxylin/light green staining. Images were acquired by Nikon Eclipse 80i microscope with a 4× 0.2 NA objective. Atherosclerotic plaque sizes were quantified using Fiji software (NIH) and represented as average of all sections in each mouse.

### Immunofluorescence

Immunofluorescent staining of 5 μm aortic root section was performed as previously described^19^. Briefly, aortic sections were stained overnight at 4 °C with primary antibodies specific to mouse antigens: hamster anti-CD11c (HL3, BD Bioscience), rat anti-CD11b-FITC (M1/70, BD Bioscience), rat anti-CD3 (17A2, Biolegend) followed by staining with secondary antibodies for 1 hour at room temperature (RT): goat anti-FITC Alexa Fluor 488 (Molecular Probes), goat anti-rat IgG Alexa Fluor 568 (Molecular Probes), and goat anti-hamster IgG DyLight 649 (Jackson Immunoresearch). MHCII was stained by anti-MHCII-PE (M5/114.15.2; eBioscience) Sections were counterstained with DAPI and embedded in Prolong Gold. Images were acquired on a Leica SP8 DM6000 inverted confocal microscope using HCX PLAPO 20x and 40x oil-immersion objectives at 405 nm, 488 nm, 563 nm and 633 nm excitation wavelength. Imaris Software was employed to adjust brightness and one-step smoothing on all images in parallel.

### RNA Isolation and Gene Expression

The tissues (aorta, spleen or paraaortic lymph node (paLN)) were homogenized with RNAse/DNase free 2.8mm Ceramic Beads using Omni Bead Ruptor 24 in PureZOL RNA Isolation Reagent (Bio-Rad Laboratories) followed by RNA isolation using Aurum Total RNA Fatty and Fibrous Tissue kit (Bio-Rad Laboratories) according to manufacturer’s protocol. First strand cDNA was synthetized using the iScript Reverse Transcription Supermix (Bio-Rad Laboratories). Gene expression was analyzed by SYBR green real-time polymerase chain reaction (Bio-Rad Laboratories) using primers for L-32, VCAM-1, ICAM-1, F4-80, MHCII, IFNγ, TNF-α, IL-17A, IL-6, IL-1α, IL-1β, CCL2, CCL5, P-selectin, E-selectin, PECAM-1. For each gene the reaction was performed in duplicate and mean value was used for calculations. Gene expression was normalized to L-32 expression. Data were analyzed using Prism statistical software (GraphPad).

### Flow Cytometry Analysis

Cells from the aorta, spleen and paLN were isolated as described before^19^. Briefly, *Apoe* ^−*/*−^ *Il27ra* ^+*/*−^ or *Apoe* ^−*/*−^ *Il27ra* ^−*/*−^ mice were euthanized by CO_2_ inhalation. The aortas were perfused with 30 ml of PBS containing 2% heparin and isolated under the dissection microscope. Collected aortas, spleens or paLNs were cut into small pieces followed by digesting in 2 ml of enzymatic cocktail, containing 450 U/ml collagenase type I, 250 U/ml collagenase type XI, 120 U/ml hyaluronidase type I, 120 U/ml DNAse I (all enzymes from Sigma) in 1x HBSS and incubated in a shaker at 37 °C for 55 min. Obtained cell suspension was stained with following antibodies: CD45-PerCP (30-F11; BioLegend), CD11b-eFluor 450 (M1/70; eBioscience), CD11c-APC (N418; eBioscience), MHCII-Alexa Fluor 700 (M5/114.15.2; eBioscience), TCRβ-eFluor 780 (H57-597; eBioscience), CD69-PE-Cy7 (H1.2F3; eBioscience), B220-FITC (RA3-6B2; eBioscience), CD4-APC (GK1.5; eBioscience), CD8-PE (53-6.7; eBioscience) and LIVE/DEAD Yellow fixable dye (Invitrogen) and analyzed by flow cytometry (LSRII, BD Biosciences). Obtained data were analyzed using FlowJo software.

### Adoptive transfer

CD45.1^+^ monocytes were isolated from spleen and peripheral blood of B6/CD45.1 mice using EasySep negative selection mouse monocyte Enrichment Kit (StemCell Tech) according to manufacturer’s protocol. 1.5 × 10^6^ cells were injected i.v. into *Apoe* ^−*/*−^ *Il27ra* ^−*/*−^ or *Apoe* ^−*/*−^ *Il27* ^+*/*−^ mice fed with WD for 7 weeks. Monocyte accumulation to the aortas was assessed by flow cytometry 48 hours after cell transfer.

### Cell sorting, labeling and antigen presentation in explanted aorta

Isolation of aortas and T cells were performed as previously described^9^. Briefly, CD4^+^ T cells were purified from spleen and paLN using Robosep negative selection kit (StemCell Technology) and labeled for 10 min at 37 °C with 2.5 µM SNARF (Molecular Probes). Aortas were surgically removed from *Apoe* ^−*/*−^ or *Apoe* ^−*/*−^ *Il27ra* ^−*/*−^ mice fed with WD for 16 weeks and incubated with 5 × 10^5^ CD4^+^ T cells obtained from spleens and paLNs of *Apoe* ^−*/*−^ *Il27ra* ^−*/*−^ mice fed WD for 16 weeks. T cells were incubated with the explanted aorta for 12 hours in 750 μl of complete RPMI 1640 media without any additional stimulation. In preparation of live image acquisition, the ends of each aorta were glued to a coverslip with Histoacryl glue (TissueSeal LLC), put in a Petri dish, maintained at 37 °C and superfused with RPMI medium 1640 without phenol red (Invitrogen) bubbled with a gas mixture containing 95% O_2_ and 5% CO_2_.

### Two-photon microscopy

Two-photon imaging was performed using a DM 6000 upright microscope with 4 non-descanned detectors (Leica Microsystems) and a Chameleon Ultra Ti: Sapphire laser (Coherent) tuned at 900 to 1000 nm for acquisition using a water-dipping objective Olympus XLUMPLFL 20XW, NA0.95. Emitted fluorescence was split with 2 dichroic mirrors (560 nm and 593 nm) and passed through filters (Semrock) 535/22 nm, 585/40 nm and 624/40 nm. To label CD4 T cells *in vivo* 10 μg of CD4-PE (RM4-5) antibody was administered to anesthetized mouse. CD4-PE was excited using confocal 543 nm laser. Typically, 10 to 20 *z*-planes spaced 10 to 15 μm apart were acquired at 512 × 512 pixels every 1 minute. The sequential acquisition with combination of 2 photon and confocal lasers was used to visualize YFP^+^ and PE^+^ cells.

### Cell tracking

The Imaris software was used to process 3D video data by detecting cells in each fluorescence channel and creating tracks by linking the detected cells over time. Tracks were manually edited to improve accuracy. The Imaris software was used to calculate interaction duration and cell velocities.

### Cytokine and chemokine protein analysis

Cell suspensions obtained from digested aortas were incubated with plate bound anti-CD3 and anti-CD28 antibodies to stimulate T cells activation for 48 h in complete RPMI 1640 media. Supernatants were collected and cytokine secretion was measured by mouse Procarta 17-Plex cytokine array (eBioscience) on MagPix instrument (Luminex) according to manufacturer’s instructions. Because 17-plex also included myeloid cell cytokines and chemokines, their production was also detected from the same supernatant.

### Statistical analysis

Data were analyzed using Prism software (GraphPad). Student’s 2-tailed T-test and Wilcoxon signed-rank test was used to compare fold induction of gene expression by real-time PCR. Data are expressed as mean ± SEM. *p < 0.05, ** p < 0.01, *** p < 0.001. P values < 0.05 was considered significant.

## Electronic supplementary material

supplementary figures and legends

Supplementary-movie1

Supplementary-movie2

Supplementary-movie3

Supplementary movie4
