# Supplementary material for: IL-27R signaling controls myeloid cells accumulation and antigen-presentation in atherosclerosis
Source: Sci Rep. 2017 May 23;7:2255. doi: 10.1038/s41598-017-01828-8 (PMC5442117; doi:10.1038/s41598-017-01828-8)
Supplement: Supplementary file 1 — supplementary figures and legends [file 41598_2017_1828_MOESM1_ESM.pdf]

Supplementary Materials

IL-27R signaling controls myeloid cells accumulation and antigen-presentation in atherosclerosis

Iuliia O Peshkova[1], Aliia R Fatkhullina[1], Zbigniew Mikulski[2], Klaus Ley[2] and Ekaterina K Koltsova[1,3]

Supplementary Figure Legends:

Supplementary Fig S1. Lipid profile and peripheral blood leukocytes count.

Body weight of *Apoe$^{-/-}$Il27ra$^{+/-}$* (n=9) or *Apoe$^{-/-}$Il27ra$^{-/-}$* (n=9) mice fed with WD for 7 weeks **(A)** or 18 weeks **(B)**. **(C)** Serum Lipid profile of *Apoe$^{-/-}$Il27ra$^{+/-}$* (n=5) or *Apoe$^{-/-}$Il27ra$^{-/-}$* (n=5) mice fed with WD for 18 weeks. **(D)** Leukocytes, monocytes, neutrophils and lympocytes count was evaluated by VetScan in blood of *Apoe$^{-/-}$Il27ra$^{+/-}$* (n=6-9) or *Apoe$^{-/-}$Il27ra$^{-/-}$* (n=6-9) mice fed with WD for 7 wks.

Supplementary Fig S2. Increased expression of chemokines in the spleen and paLN of *Apoe$^{-/-}$Il27ra$^{-/-}$* mice. **(A, B)** CCL2 and CCL5 were measured by bead array in supernatants of splenic or paLN cell suspension obtained from *Apoe$^{-/-}$Il27ra$^{+/-}$* (n=5) or *Apoe$^{-/-}$ Il27ra$^{-/-}$* (n=5) mice fed with WD for 7 weeks **(A)** or 18 weeks **(B)**, stimulated with anti-CD3/anti-CD28 for 48 hours. Data are represented as mean ± SEM. *P <0.05, ** P<0.01, *** P<0.001. P values <0.05 was considered significant.

Supplementary Fig S3. Enchanced accumulation of immune cells in aortas of *Apoe$^{-/-}$ Il27ra$^{-/-}$* mice with advanced atherosclerosis. Live CD45^{+} cells from aortas of *Apoe$^{-/-}$Il27ra$^{+/-}$* or *Apoe$^{-/-}$Il27ra$^{-/-}$* mice fed with WD for 18 weeks were stained for CD45^{+}, CD11b^{+}, CD11c^{+} and TCRβ^{+}. Percentage **(left)** and cell number **(right)** **(A-C)** of live CD45^{+}, CD11b^{+}, CD11b^{+}CD11c^{+} and CD11c^{+} cells, TCRβ^{+} T cells in aortas of *Apoe$^{-/-}$Il27ra$^{+/-}$* or *Apoe$^{-/-}$Il27ra$^{-/-}$* mice fed with WD for 18 weeks was quantified by flow cytometry. Data are mean ± SEM from at least 3

independent experiments. **(D)** Confocal images demonstrate localization of $CD11b^{+}CD11c^{-}$, $CD11b^{+}CD11c^{+}$, $CD11b^{-}CD11c^{+}$ myeloid cells in aortic roots of *Apoe*$^{-/-}$*Il27ra*$^{-/-}$ or *Apoe*$^{-/-}$*Il27ra*$^{+/-}$ mice fed with WD for 7 weeks (early lesions) or 18 weeks (advanced lesions).

Supplementary Fig S4. Increased MHCII and CD69 expression in the spleen and paLN of *Apoe*$^{-/-}$ *Il27ra*$^{-/-}$ mice. Expression of MHCII by $CD11b^{+}CD11c^{+}$ and $CD11c^{+}$ cells in the spleen **(A)** or paLN **(B)** of *Apoe*$^{-/-}$*Il27ra*$^{+/-}$ or *Apoe*$^{-/-}$*Il27ra*$^{-/-}$ mice fed with WD for 7 weeks. **(C)** Expression of CD69, a marker of T cell activation, by $CD4^{+}$ T cells in the spleen of *Apoe*$^{-/-}$ *Il27ra*$^{+/-}$ or *Apoe*$^{-/-}$*Il27ra*$^{-/-}$ mice fed with WD for 7 weeks.

Supplementary Fig S5. Increased expression of pro-inflammatory cytokines in the spleen and paLN of *Apoe*$^{-/-}$*Il27ra*$^{-/-}$ mice. Production of pro-inflammatory cytokines in *Apoe*$^{-/-}$*Il27ra*$^{+/-}$ or *Apoe*$^{-/-}$*Il27ra*$^{-/-}$ mice at different stages of atherosclerosis was measured by multiplex cytokines array in supernatants of splenic and paLN cell suspension obtained from *Apoe*$^{-/-}$*Il27ra*$^{+/-}$ (n=5) or *Apoe*$^{-/-}$*Il27ra*$^{-/-}$ (n=5) mice fed with WD for 7 **(A,B)** or 18 weeks (**C,D)**, stimulated with anti-CD3/anti-CD28 for 48 hours. Data are represented as mean ± SEM. *$P < 0.05$, ** $P<0.01$, *** $P<0.001$. P values <0.05 was considered significant.

Supplemental Movie Legends

Supplementary Movie S1. Localization of $CD11c^{YFP+}$ APC in the atherosclerotic arterial wall of *$Apoe^{-/-}CD11c^{YFP+}$* mice. YFP^{+} APC (green); second harmonic (blue). Representative movie of at least 5 independent experiments.

Supplementary Movie S2. Localization of $CD11c^{YFP+}$ APC in the atherosclerotic arterial wall of *$Apoe^{-/-}Il27ra^{-/-}CD11c^{YFP+}$* mice. YFP^{+} APC (green); second harmonic (blue). Representative movie of at least 5 independent experiments.

Supplementary Movie S3. Migration and interaction of $CD11c^{YFP+}$APC (green) in the aorta of *$Apoe^{-/-}CD11c^{YFP+}$* mice with *$Apoe^{-/-}Il27ra^{-/-}$* $CD4^{+}$ T cells, isolated from *$Apoe^{-/-}Il27ra^{-/-}$* mice fed with WD for 16 weeks and labeled with SNARF (red). No antigen added. Maximum intensity projection along zaxis. Adventitial localization of $CD11c^{YFP+}$ APC and $CD4^{+}$ T cells. Representative movie of 4 independent experiments.

Supplementary Movie S4. Migration and interaction of $CD11c^{YFP+}$APC (green) in the aorta of *$Apoe^{-/-}Il27ra^{-/-}CD11c^{YFP+}$* mice with *$Il27ra^{-/-}Apoe^{-/-}$* $CD4^{+}$ T cells, isolated from *$Il27ra^{-/-}Apoe^{-/-}$* mice fed with WD for 16 weeks and labeled with SNARF (red). No antigen added. Maximum intensity projection along z axis. Adventitial localization of $CD11c^{YFP+}$ APC and $CD4^{+}$ T cells. Representative movie of 4 independent experiments.

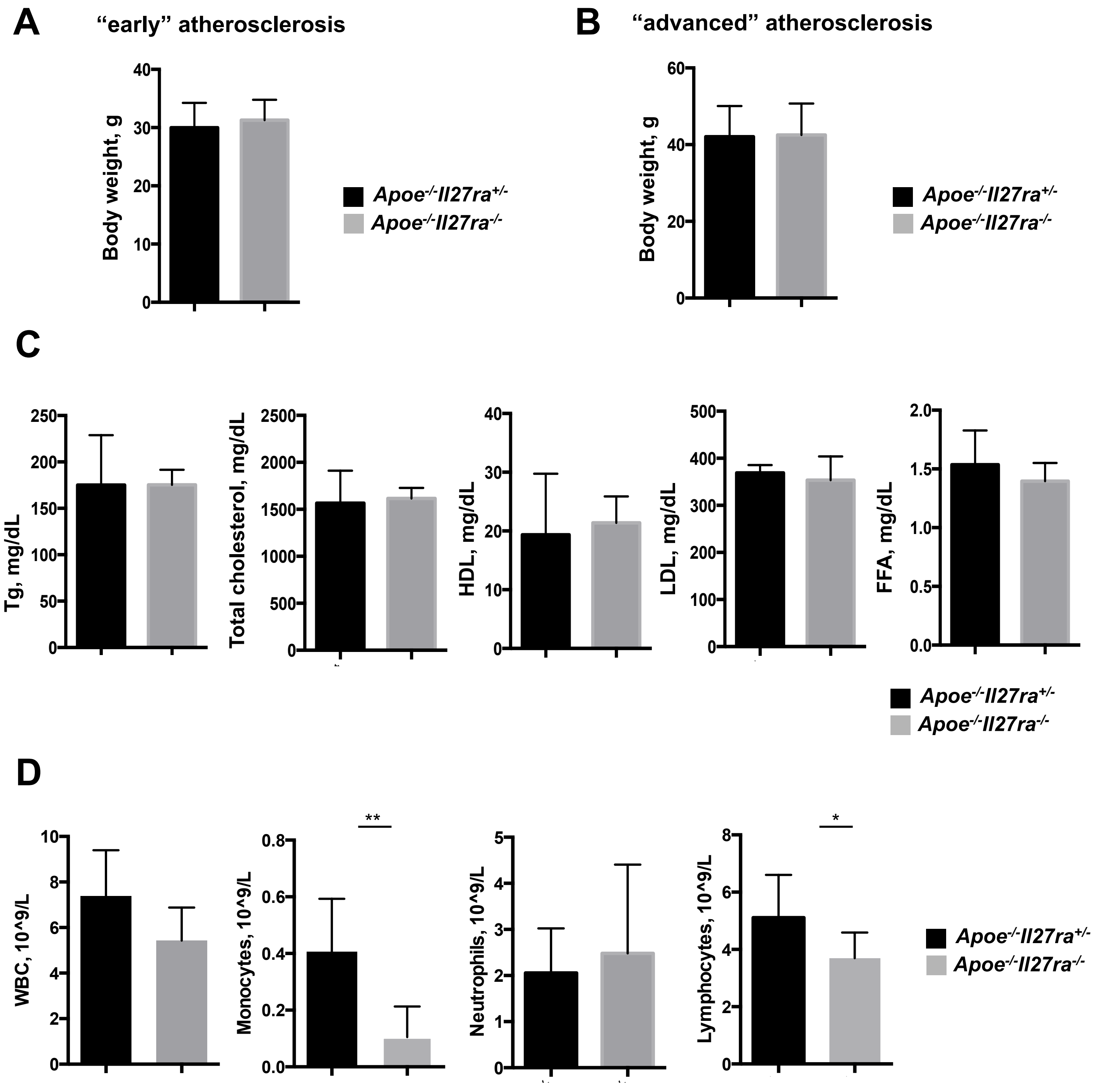

Supplementary Figure S1. Lipid profile and blood leukocytes count.

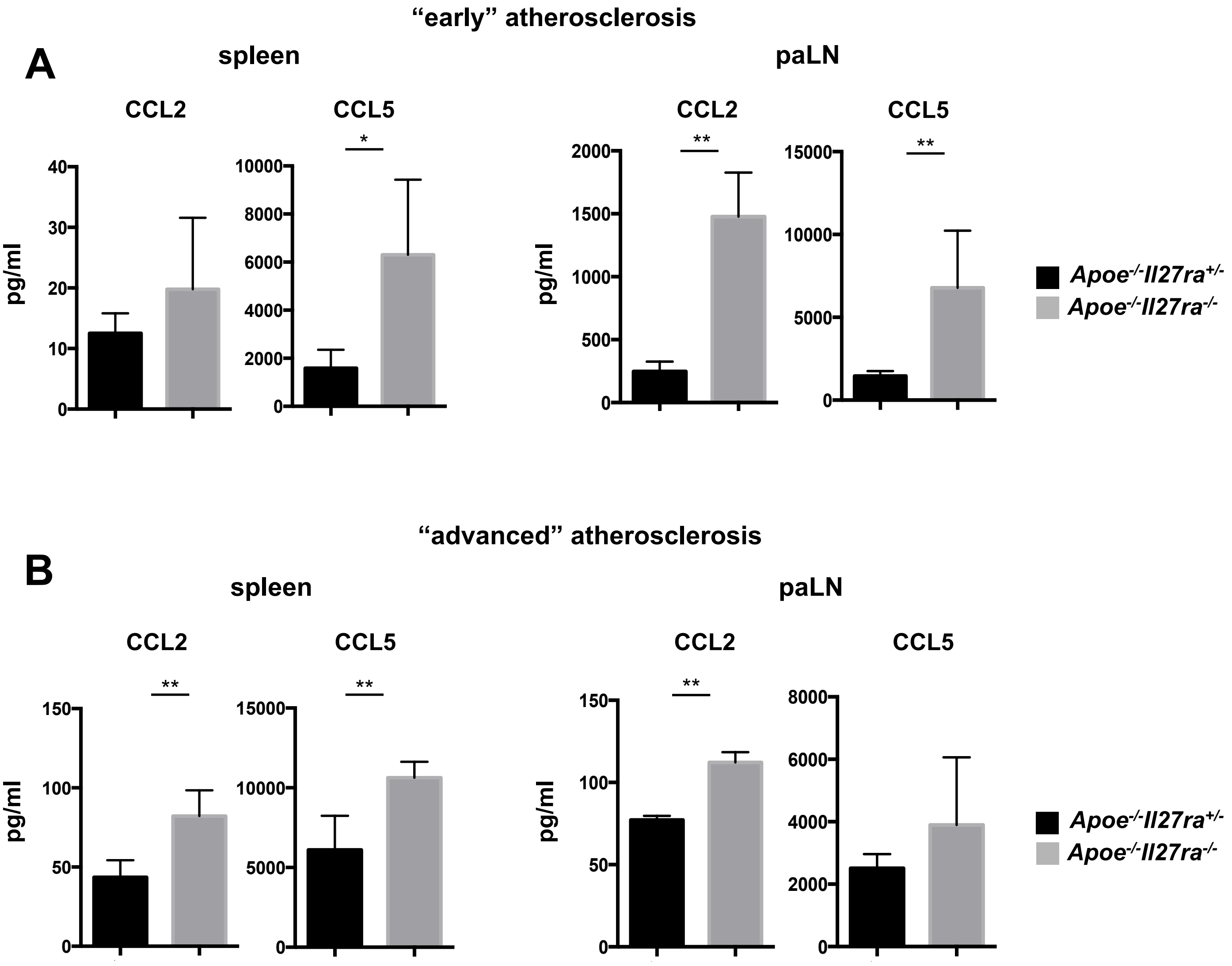

Supplementary Figure S2. Increased expression of chemokines in the spleen and paLN of *Apoe$^{-/-}$ Il27ra$^{-/-}$* mice.

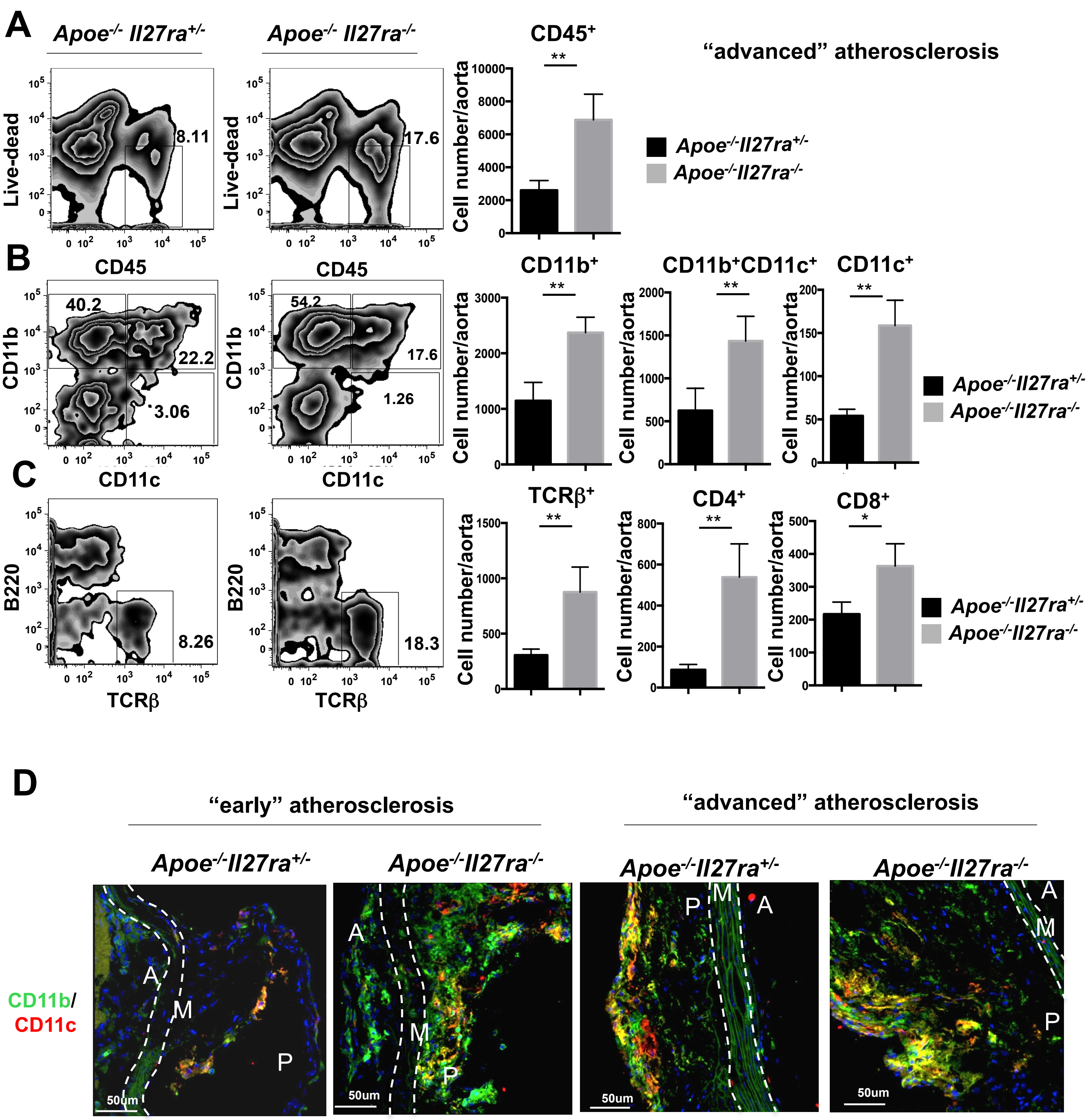

Supplementary Figure S3. Enhance accumulation of immune cells in aortas of *Apoe-/-Il27ra-/-* mice with advanced atherosclerosis

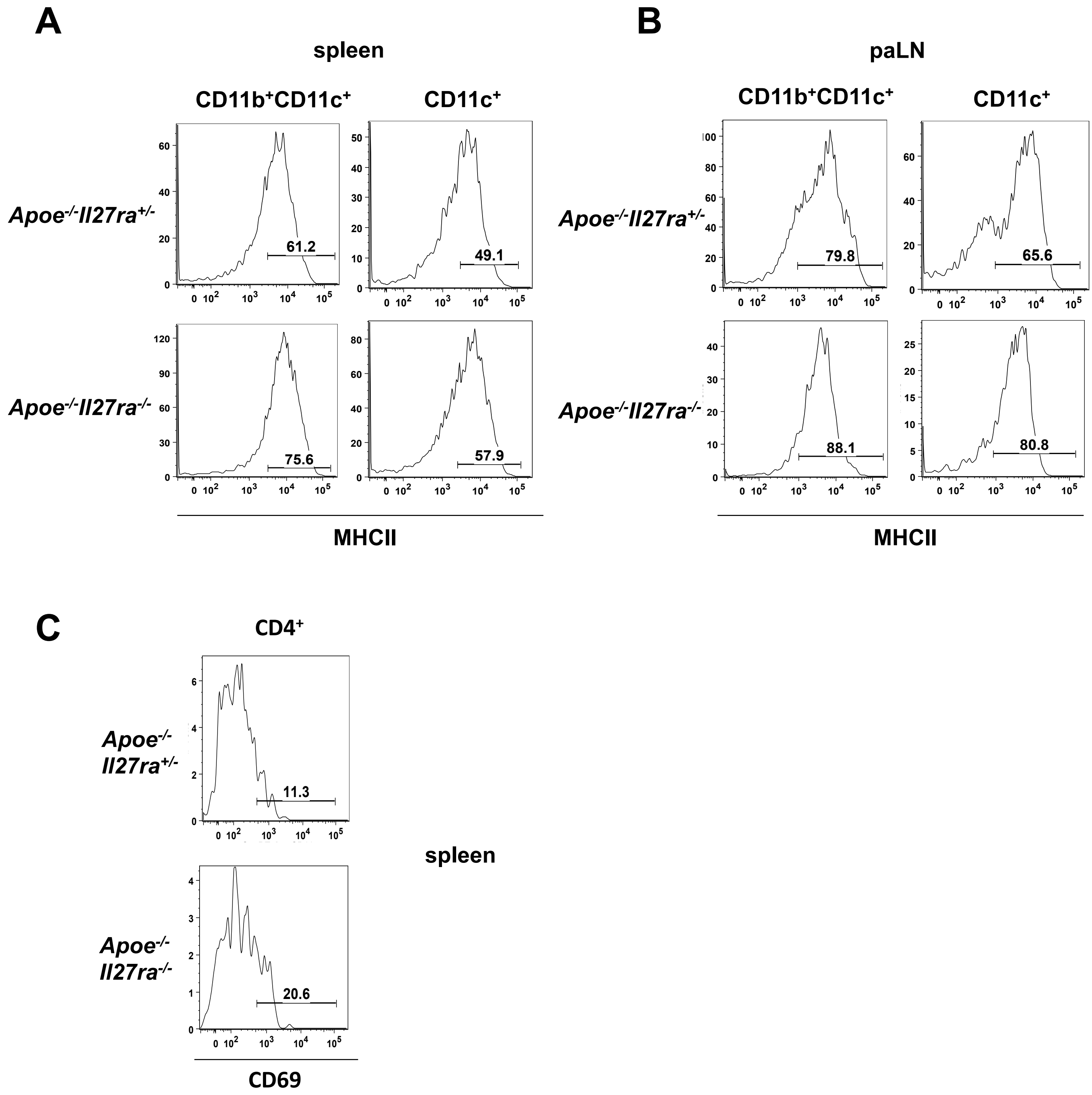

Supplementary Figure S4. Increased MHCII and CD69 expression in spleen and paLN of *Apoe$^{-/-}$ Il27ra$^{-/-}$* mice.

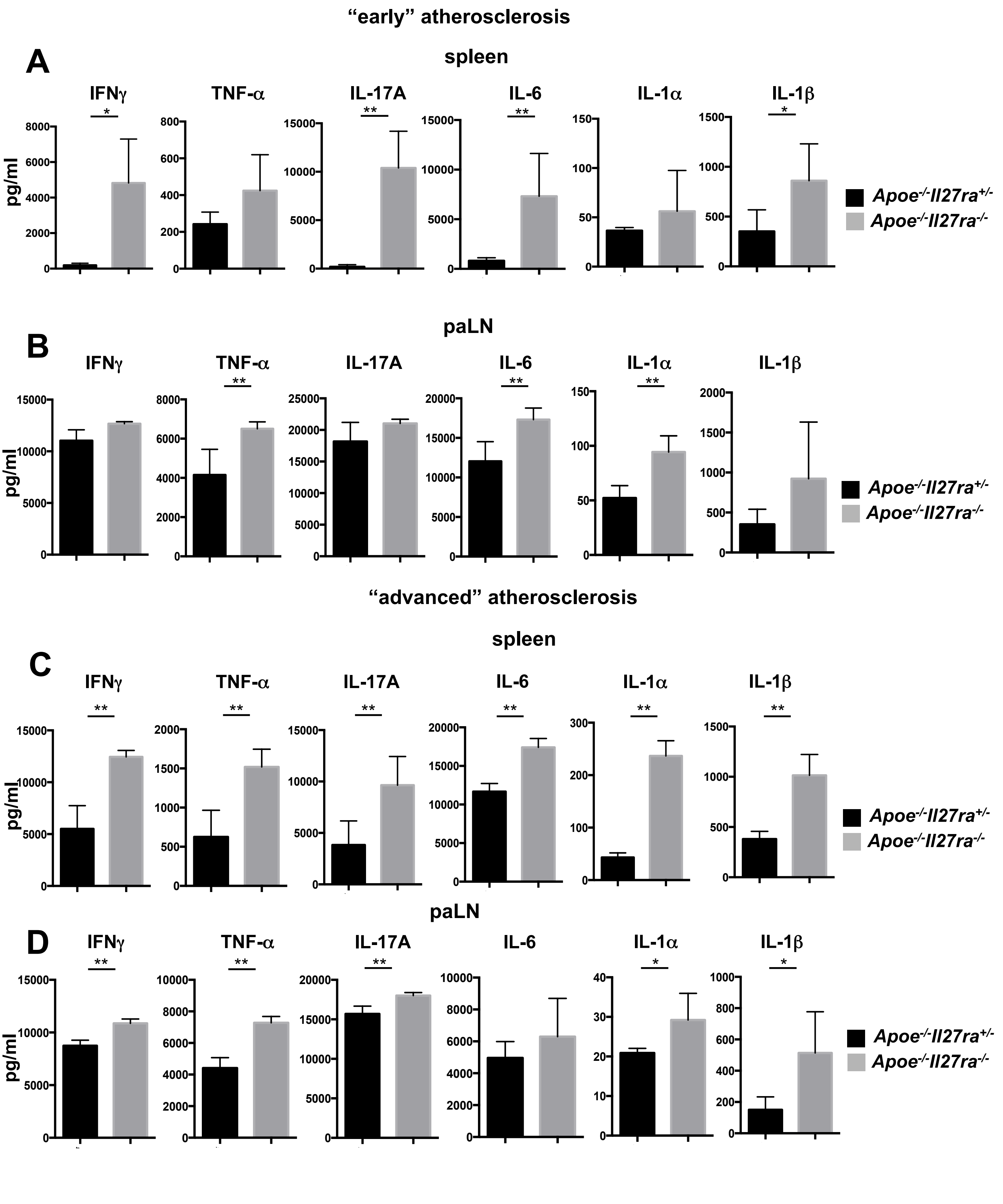

Supplementary Figure S5. Increased expression of pro-inflammatory cytokines in the spleen and paLN of *Apoe$^{-/-}$Il27ra$^{-/-}$* mice.
